# Factors affecting the use of biosecurity measures for the protection of ruminant livestock and farm workers against infectious diseases in central South Africa

**DOI:** 10.1111/tbed.14525

**Published:** 2022-04-05

**Authors:** Veerle Msimang, Melinda K. Rostal, Claudia Cordel, Catherine Machalaba, Stefano Tempia, Whitney Bagge, Felicity J. Burt, William B. Karesh, Janusz T. Paweska, Peter N. Thompson

**Affiliations:** ^1^ Epidemiology Section Department of Production Animal Studies Faculty of Veterinary Science University of Pretoria Onderstepoort South Africa; ^2^ Centre for Emerging Zoonotic and Parasitic Diseases National Institute for Communicable Diseases of the National Health Laboratory Service Sandringham South Africa; ^3^ EcoHealth Alliance, New York New York NY USA; ^4^ Institute of Biodiversity, Animal Health and Comparative Medicine College of Medical, Veterinary and Life Sciences University of Glasgow Glasgow UK; ^5^ ExecuVet (Pty) LTD Bloemfontein South Africa; ^6^ Centre for Respiratory Diseases and Meningitis National Institute for Communicable Diseases of the National Health Laboratory Services Johannesburg South Africa; ^7^ Faculty of Health Sciences School of Public Health University of the Witwatersrand Johannesburg South Africa; ^8^ Division of Virology National Health Laboratory Service Universitas Bloemfontein South Africa; ^9^ Division of Virology Faculty of Health Sciences University of the Free State Bloemfontein South Africa; ^10^ Centre for Viral Zoonoses University of Pretoria Pretoria South Africa

**Keywords:** biosecurity, farmers, ruminant production, South Africa, zoonoses

## Abstract

Biosecurity measures have been introduced to limit economic losses and zoonotic exposures to humans by preventing and controlling animal diseases. However, they are implemented on individual farms with varying frequency. The goal of this study was to evaluate which biosecurity measures were used by farmers to prevent infectious diseases in ruminant livestock and to identify factors that influenced these decisions. We conducted a survey in 264 ruminant livestock farmers in a 40,000 km^2^ area in the Free State and Northern Cape provinces of South Africa. We used descriptive statistics, to characterize biosecurity measures and farm attributes, then multivariable binomial regression to assess the strength of the association between the attributes and the implementation of biosecurity measures including property fencing, separate equipment use on different species, separate rearing of species, isolation of sick animals, isolation of pregnant animals, quarantine of new animals, animal transport cleaning, vaccination, tick control and insect control. Ninety‐nine percent of farmers reported using at least one of the 10 biosecurity measures investigated (median [*M*]: 6; range: 0–10). The most frequently used biosecurity measures were tick control (81%, 214 out of 264), vaccination (80%, 211 out of 264) and isolation of sick animals (72%, 190 out of 264). More biosecurity measures were used on farms with 65–282 animals (*M*: 6; odds ratio [OR]: 1.52) or farms with 283–12,030 animals (*M*: 7; OR: 1.87) than on farms with fewer than 65 animals (*M*: 4). Furthermore, farmers who kept two animal species (*M*: 7; OR: 1.41) or three or more species (*M*: 7) used more biosecurity measures than single‐species operations (*M*: 4). Farmers with privately owned land used more biosecurity measures (*M*: 6; OR: 1.51) than those grazing their animals on communal land (*M*: 3.5). Farms that reported previous Rift Valley fever (RVF) outbreaks used more biosecurity measures (*M*: 7; OR: 1.25) compared with farms without RVF reports (*M*: 6) and those that purchased animals in the 12 months prior to the survey (*M*: 7; OR: 1.19) compared with those that did not (*M*: 6). When introducing new animals into their herds (*n* = 122), most farmers used fewer biosecurity measures than they did for their existing herd: 34% (41 out of 122) used multiple biosecurity measures like those of vaccination, tick control, quarantine or antibiotic use, whereas 36% (44 out of 122) used only one and 30% (37 out of 122) used none. Certain farm features, primarily those related to size and commercialization, were associated with more frequent use of biosecurity measures. Given the variation in the application of biosecurity measures, more awareness and technical assistance are needed to support the implementation of a biosecurity management plan appropriate for the type of farm operation and available resources.

## 1 INTRODUCTION

Agriculture plays a key role in the growth of African countries and there is an immense need for stability and improved productivity in this sector (Audibert, 2010). Sustainability issues have been raised in the livestock production subsector as the global demand for protein‐rich diets is forecasted to continue to increase (Delport et al., 2017; Department of Agriculture Forestry and Fisheries Republic of South Africa, 2019; Organisation for Economic Co‐operation Development and the Food and Agriculture Organization of the United Nations, 2020). The livestock sector is a major employer and contributes substantially to food security in South Africa (Meissner et al., 2013). Livestock farming is a critical economic driver and provides the sustenance for most non‐metropolitan towns and rural communities (Meissner et al., 2013). In many rural areas, the use of pasture is a common herd management practice for ruminants (Palisson et al., 2017). The most limiting factors to pasture‐based livestock production in South Africa have been animal diseases, land rights and access, inadequate knowledge of livestock and pasture management and climate variability (Oduniyi et al., 2020). One of the most sustainable ways to protect against threats of infectious diseases and reduce their economic costs is through the implementation of biosecurity (Oliveira et al., 2017).

The World Organisation for Animal Health (OIE) Terrestrial Animal Health Code (2019) defines biosecurity as ‘the set of managerial and physical measures designed to reduce the introduction, establishment and spread of animal diseases, infections or infestations to, from and within an animal population’. Livestock farmers are responsible for and directly benefit from the continued implementation of biosecurity on their farm (Manuja et al., 2014). Prioritizing animal health and welfare improves productivity, enhances resilience to climate change and natural disasters, such as drought and has economic benefits (Vallat, 2015; Lubroth et al., 2017). A robust biosecurity program improves animal welfare by keeping more animals healthy and resistant to environmental factors; it also serves as the first line of defence by detecting diseases early and limiting disease spread within the farm (Kriel, 2018), reduces the costs of treating diseases, helps to ensure production of high‐quality, safe, nutritious products and improves the chances of running a successful business and remaining in the agricultural industry (Sinclair et al., 2019). In addition to protecting animal health and its economic benefits, when biosecurity measures are used to limit infectious diseases in livestock, they also directly reduce the risk of zoonotic pathogen transmission to humans and can help to inform specific public health measures (Kimman et al., 2013; Layton et al., 2017). The implementation of farm biosecurity measures should prioritise animal diseases with adverse social, welfare and economic effects and can be designed as multi‐hazard mitigation methods (Sternberg‐Lewerin et al., 2015; Layton et al., 2017).

Standards and recommendations for a wide range of biosecurity practices for livestock production, either for general disease prevention or to minimise specific infection risks, including zoonotic risks are widely available on the internet (Waage & Mumford, 2008; African Union Inter‐African Bureau for Animal Resources, 2015; Windsor, 2017; Robertson, 2019; Van Der Merwe, 2020). Recommended on‐farm biosecurity measures include: animal hygiene, sanitation, restrictions on sharing and disinfection procedures for equipment, vehicles and facilities, tick and pest control, vaccination, movement controls and quarantine of newly introduced animals, preventing different groups of animals from mixing, culling of diseased animals, protocols for the handling and treatment of infected animals or contaminated products, feed management, facility and vehicle maintenance and protocols for handling manure and disposing of carcasses.

There are some published studies on the knowledge, attitudes and practices of farmers regarding animal health care and biosecurity in Africa (Simela, 2012; Oladele et al., 2013; Wolff et al., 2019). One recent study examined the determinants of animal health care practices that included vaccination, external and internal parasite control, quarantine of new and isolation of sick animals, restricted access and supplementary feeding in South Africa (Mdlulwa et al., 2021); however, the majority of studies that analyze biosecurity measures used by farmers have been conducted in high income countries (Heffernan et al., 2008; Dorea et al., 2010; Sarrazin et al., 2014; Renault et al., 2018; Gunther et al., 2019). The benefits of biosecurity in terms of productivity and profitability have often focused on specific production and management systems, a single biosecurity measure or the prevention of one disease only (Gunn et al., 2008; Laanen et al., 2013; Merrill et al., 2019). Studies on pasture‐grazed systems are often neglected. It is often believed that the choice to implement biosecurity measures by individual farmers is associated with economic constraints and infectious disease awareness (Niemi et al., 2016; Merrill et al., 2019). However, awareness is not necessarily the limiting constraint on practicing biosecurity interventions and some farmers would prefer to pay for treatment rather than prevention and standard biosecurity practices (Dione et al., 2020). Challenges to adopting biosecurity measures for pasture‐based systems include financial cost, resulting in fewer and only certain specific measures being implemented, and lack of research (Niemi et al., 2016). Some biosecurity interventions, for example, culling, may cause particular hardship for the livestock owners (Fraser, 2018).

The lack of or inefficient implementation of biosecurity measures and the resultant disease on the farm have downstream effects on all livestock owners who are connected geographically or through market chains. If an outbreak occurs due to an individual landowner's negligence, the surrounding land users are likely to suffer significant financial consequences (Kristensen & Jakobsen, 2011; Knight‐Jones & Rushton, 2013).

Data on current, local biosecurity practices are important for veterinary professionals and government in order to support farmers in biosecurity decision‐making and identify areas for targeted education to increase the effectiveness of disease control and surveillance. In this study, we examined which and how many biosecurity measures are being implemented by ruminant livestock farmers in the Free State and Northern Cape provinces of South Africa, and evaluated the farm characteristics associated with the likelihood of implementation of biosecurity measures.

## 2 MATERIALS AND METHODS

### 2.1 Study area

The study was conducted over an area of ∼40,000 km^2^, at 994–1794 m above sea level, in the Free State and Northern Cape provinces, between Bloemfontein (29.0852°S, 26.1596°E) and Mokala National Park (29.1659°S, 24.3197°E). The ecosystem primarily consists of tall dry Highveld grassland grazed by commercial cattle and sheep (Waldner et al., 2017). The western portion of the study area in Northern Cape is dry savannah, and the southern portion is part of the Great Karoo with grassy shrubland (Köppen & Geiger, 1936). The climate in the study area is mostly temperate semi‐arid steppe (Köppen & Geiger, 1936). This area was selected because it had been affected by large RVF outbreaks in the past.

### 2.2 Study design and sampling strategy

Two cross‐sectional surveys of farms keeping ruminants were conducted during October 2015–August 2016 and May–November 2017. The sampling and methodology, including the study farms selection via random geographic points, were described previously by Ngoshe et al. (2020) and Msimang et al. (2019). We contacted the participating farms from the first survey for the second survey and replaced those that declined to participate again with another farm near the next random geographic point on the list. The sample size of 264 farms provided greater than 80% power to detect a count ratio of 1.2 for a binary predictor of interest, assuming a baseline count of 5 biosecurity measures and an R‐squared of 0.25 between the predictor of interest and the other covariates in the model (Supporting Information S2) (Signorini, 1991).

### 2.3 Questionnaire

Participating farmers provided informed, written consent and were administered a questionnaire which collected information on farm animals, management, husbandry practices, biosecurity measures used and environmental characteristics of the farm (Supporting Information S1). The questionnaire evaluated the use of 10 biosecurity measures by the farm: (1) maintaining fencing around the property, (2) keeping different animals species in different/divided areas on the farm, (3) having separate equipment for different species, (4) feeding, treating and working with sick animal(s) after working with healthy animals, (5) keeping pregnant animals separate from the herd, (6) quarantine of new animals before joining the existing herd, (7) cleaning and disinfecting vehicles before and after transporting animals, (8) vaccination, (9) tick control (dipping animals, pour‐ons or giving an injection) and (10) biting fly/mosquito control. We also evaluated whether the farmer used the following biosecurity measures when introducing new animals into the herd: (a) quarantine, (b) antibiotic administration, (c) vaccination, (d) tick control. Only the responses from farmers who had purchased new stock in the previous 12 months were included in the assessment of measures relating to newly introduced animals. We categorized farms based on land ownership, with farmers/farms grazing their animals on privately owned land referred to as ‘private farmers/farms’, and those grazing their animals on communal land referred to as ‘communal farmers/farms’, and their self‐identified production system (no‐cash/subsistence farming, cash livestock systems, semi‐commercial with limited marketing of farm products, commercial farms and feedlots). The ‘semi‐commercial’ and ‘cash‐sales’ were combined with ‘no‐cash sales’ production systems for analysis. The private farmers' responses to the question about the main reason for keeping the animals were limited to combinations of meat, wool and dairy, but communal farmers were allowed additional responses that were not asked to the private farmers. We created a ‘yes/no’ variable for farms that kept horses taken from the question about which other animals were reared on the farm. In addition, a count variable on number of species on the farm was created to be used in the multivariable analysis instead of including the ‘yes/no’ responses to keeping individual species of animals. Questions were asked about experience with selected pathogens (Rift Valley fever [RVF] virus and *Brucella* species). For all farmers that completed the questionnaire in both 2015–16 and 2017, the answers from the first survey were used. The questionnaire was piloted among 17 farmers located just outside the study area in May 2015. The questionnaire was administered to the farm owner or manager in English, Afrikaans or Sesotho using a tablet. At the time of consent, a farm ID number was assigned and anonymous responses were sent to an internet cloud‐based database using the Open Data Kit Application (Hartung et al., 2010). Individual identifying information was not captured by the electronic questionnaire.

### 2.4 Data analysis

The electronic questionnaire data were downloaded and cleaned using RStudio (RStudio Team, 2020). Further data cleaning/structuring was conducted in Microsoft Excel and the data were then imported into Stata 13 (StataCorp, College Station, TX, USA) for the statistical analyses.

For the descriptive analysis, we calculated percentages, medians, ranges and quartiles for each farm and/or animal characteristics, as well as the use of the 10 biosecurity measures. The Fischer's exact test was used to test whether the percentages for each biosecurity measure of private and communal farmers differed significantly. The odds ratios (OR) and 95% confidence intervals (CI) of implementing a biosecurity measure by the farmers with versus without various farm characteristics were estimated using a multivariable maximum likelihood binomial regression analysis, modelling the number of successes (measures implemented) out of the number of trials (possible measures). The number of possible measures was 10 for farmers raising multiple species but only 8 for farmers who raised only one animal species because they did not have the opportunity to implement ‘(2) keeping different animal species in different/divided areas on the farm’ and ‘(3) having separate equipment for different species’. The assumption of independence in binomial regression was validated by measuring pairwise correlations between biosecurity measures using Phi coefficient calculations $\varphi \;=\frac{ad-bc}{\sqrt{efgh}}$ where *a*–*d* are frequencies of the cells of the 2 × 2 table and *e*–*h* (*e* = *a* + *b*, *f* = *c* + *d*, *g* = *a* + *c*, *h* = *b* + *d*) the totals, with values ranging from 0.0 to +0.3 denoting little or no correlation, +0.3 to +0.7 denoting a weak positive correlation and +0.7 to +1.0 denoting a strong positive correlation (Table S1) (Simon, 2007). The final binomial regression model was obtained by manual backward elimination, starting with all independent variables that had significant (*p *< .2) univariable associations using binomial regression and continuing until all remaining variables were significant (*p *< .05), or if removal of a confounding variable resulted in >10% change in the coefficient of another variable; finally, forward stepwise selection was done to assess previously eliminated variables in order to achieve a final model of significant predictors.

## 3 RESULTS

### 3.1 Animal and farm description

**FIGURE 1:**
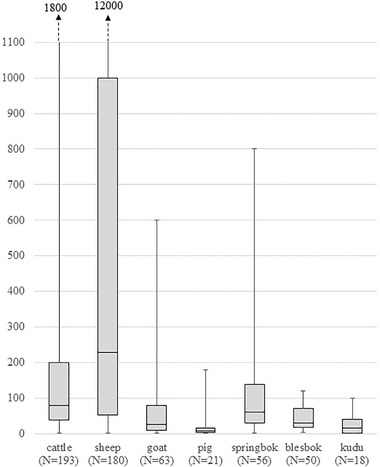
Number of animals by species kept on ruminant livestock farms in the Free State and Northern Cape, South Africa. Whiskers indicate range, box indicates median and interquartile range, *N* indicates number of farms

A total of 264 ruminant livestock farms were surveyed; 228 and 36 farms completed their first survey in 2015–16 and 2017, respectively. Of these, 32 farms used communal land, whereas 232 used privately owned land (ratio: ˜1:7). In terms of farm animal species, 73% (193 out of 263) kept cattle, 69% (180 out of 261) kept sheep, 24% (63 out of 263) kept goats and 26% (68 out of 264) kept wild antelope. A total of 52% (135 out of 261) farms kept both cattle and sheep. Animal numbers per farm (Figure 1) were highest for sheep, with a median of 229 (range: 2–12,000; *n* = 180 farms) and the median number of cattle was 80 (range: 1–1800; *n* = 193 farms). Private land ownership was significantly associated with keeping multiple species and a larger herd size.

The median area of and number of employees on the farm varied based on the land‐tenure system of the farms (private versus communal land). Private farmers owned a median of 1200 hectares of land (interquartile range [IQR]: 428–3000; range: 2—15,000) and had a median of 3 employees (range: 0–45). Most communal farmers did not have employees. Workers (with or without their families) lived on the premises at 85% (224 out of 264) of the farms. A median of 5.5 adults (IQR: 3–11; range: 1–74) and 2 children (<18 years) (IQR:0‐6; range: 0–140) lived on the farms.

Of farms surveyed, 57% (151 out of 264) raised livestock for meat products, 19% (51 out of 264) for meat and wool production and 14% (36 out of 264) for resale, whereas other purposes reported were dairy, barter, tourism or ceremonial purposes. 64% (147 out of 232) of the respondents among private farmers self‐identified as commercial, 13% (31 out of 232) were semi‐commercial, 10% (24 out of 232) had cash sales, 9% (21 out of 232) were feedlots and 4% (nine out of 232) non‐cash sale farms. Of respondents among communal farmers, 75% (24 out of 32) self‐identified primarily as keeping livestock for cash sales, 19% (six out of 32) for non‐cash sale needs and 6% (two out of 32) were semi‐commercial.

### 3.2 Animal movements and purchases

Multiple animal species were permitted to interact at certain or all times on 51% (119 out of 232) of private farms and 72% (23 out of 32) of communal farms. Cattle and sheep were allowed to intermix on 63% (82 out of 130) of the private farms. Only 10 communal farmers raised both cattle and sheep, and seven of them allowed the two species to intermingle. Animals were grazed in areas where wildlife could pass through on 47% (109 out of 232) of the private farms, whereas only 25% (eight out of 32) of communal farmers reported interaction of their livestock with wildlife. Cattle and sheep were pastured at night on 87% (159 out of 182) and 62% (106 out of 172) of private farms, respectively, whereas on communal farms cattle in only 29% (four out of 14) of the farms and sheep in 13% (four out of 32) of the farms, with the majority keeping their animals in an open corral overnight. On 19% (43 out of 232) of private farms, the animals were able to interact with the animals of a neighbouring farm, a median of once a week (IQR:1‐5; range: 1–7); the nearest neighbouring farm was a median of 3 km away (IQR: 1–5; range: 80 m to 20 km). This contrasts with the 59% (19 out of 32) of communal farmers that reported their animals were allowed to contact neighbouring animals daily (*M*: 7/week; IQR: 7–7; range: 3–7). An 81% (189 out of 232) of private farms used a rotational grazing management system and the median number of times per year they rotated the grazing area was four (IQR: 3–6; range: 1–52). Among communal farmers, only 16% (five out of 32) of owners used a rotational grazing system. On 19% (six out of 32) of communal farms and 6% (14 out of 232) of private farms, livestock were allowed to enter the house or compound housing area.

In the 12 months prior to the survey, 50% (115 out of 232) of private farms reported purchasing animals and bringing a variety of new animal species onto the farm. Animal acquisition was less common among communal farmers, with only 22% (seven out of 32) of farms doing so; three farms purchased cattle, two goats, one cattle and goats and one pigs.

### 3.3 Animal losses from abortion

Abortions in farm animals were reported on 25% of farms (67 out of 264) in the 3 months preceding the survey, with similar proportions on private and communal farms. Abortions in sheep were reported by 60% (40 out of 67) of farms and 42% (28 out of 67) of farms reported abortions in cattle, whereas low rates were reported in goats, springbok, blesbok and gemsbok. The proportion of animals aborting varied from 0.01 to 17% (one out of 13,000–one out of six) in farms with only sheep to 0.2–50% (one out of 550–one out of two) in farms that only kept goats. Fifty‐seven percent (38 out of 67) of respondents said they buried or burned the abortus, whereas 43% (29 out of 67) said they left the abortus untouched.

### 3.4 Reported outbreaks of selected agents

We found that 30% (95% CI: 24–36%; 69 out of 232) of private farmers and 9% (95% CI: 3–27%; three out of 32) of communal farmers reported experiencing a possible RVF outbreak in the past. Among the private farmers, 10% (95% CI: 7–15%; 23 out of 232) reported animal cases of brucellosis in the past, of which 87% (20 out of 23) reported it in cattle, with the remainder in sheep and goats. Farmers reported their most recent brucellosis outbreak from 1980 to 2017 (*n* = 16). No communal farmers were aware of any previous brucellosis cases amongst their livestock.

### 3.5 Routine use of biosecurity measures on the farm

**FIGURE 2:**
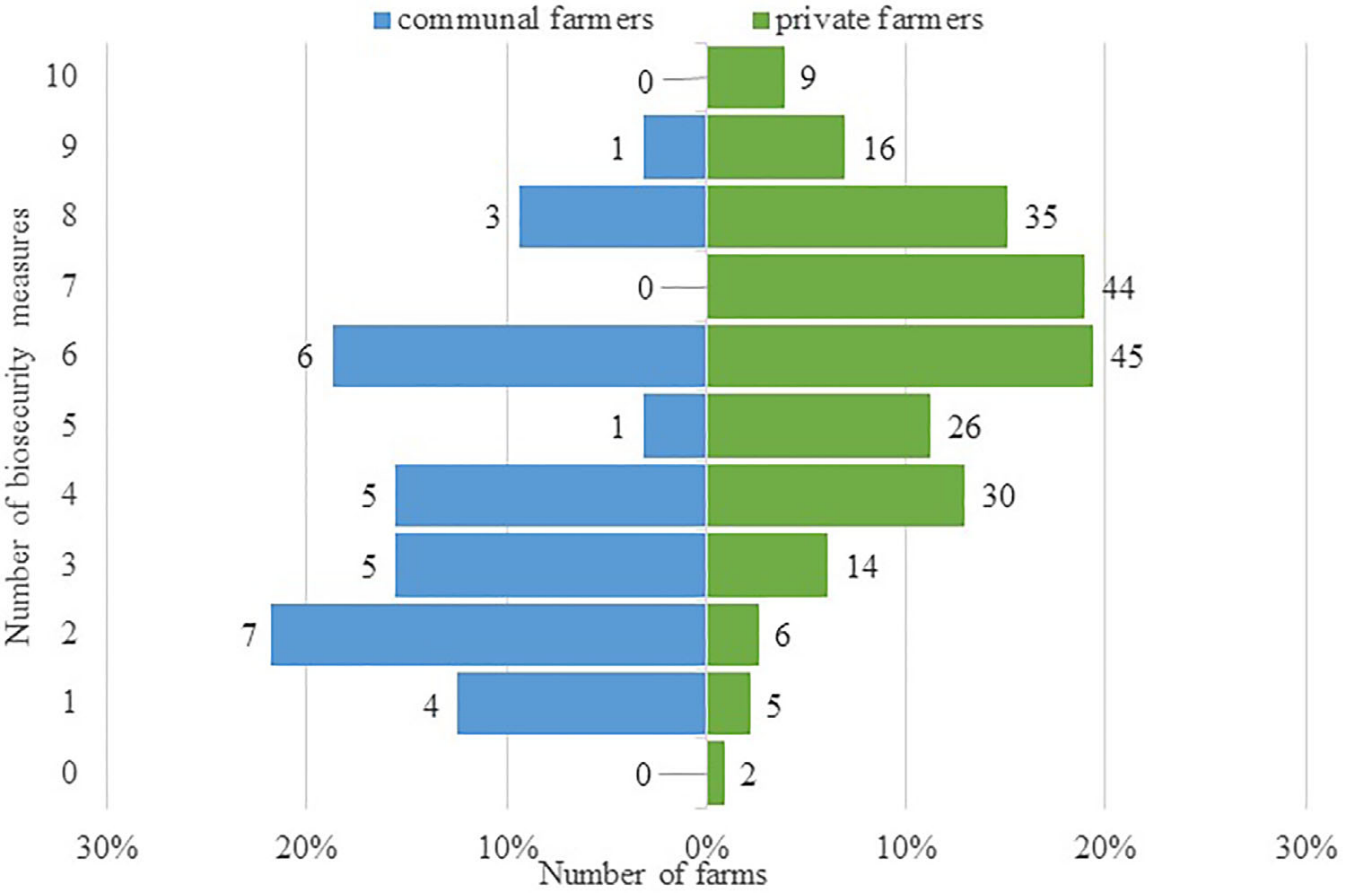
Number of specified biosecurity measures used on ruminant livestock farms in Free State and Northern Cape, South Africa. Measures were: maintaining fencing around the property, keeping different animal species in different areas on the farm, having separate equipment for different species, feeding, treating and working with sick animals after working with healthy animals, keeping pregnant animals separate from herd, quarantining of new animals before joining the herd, cleaning and disinfecting vehicles before and after transporting animals, vaccination, tick control and biting fly/mosquito control

**FIGURE 3:**
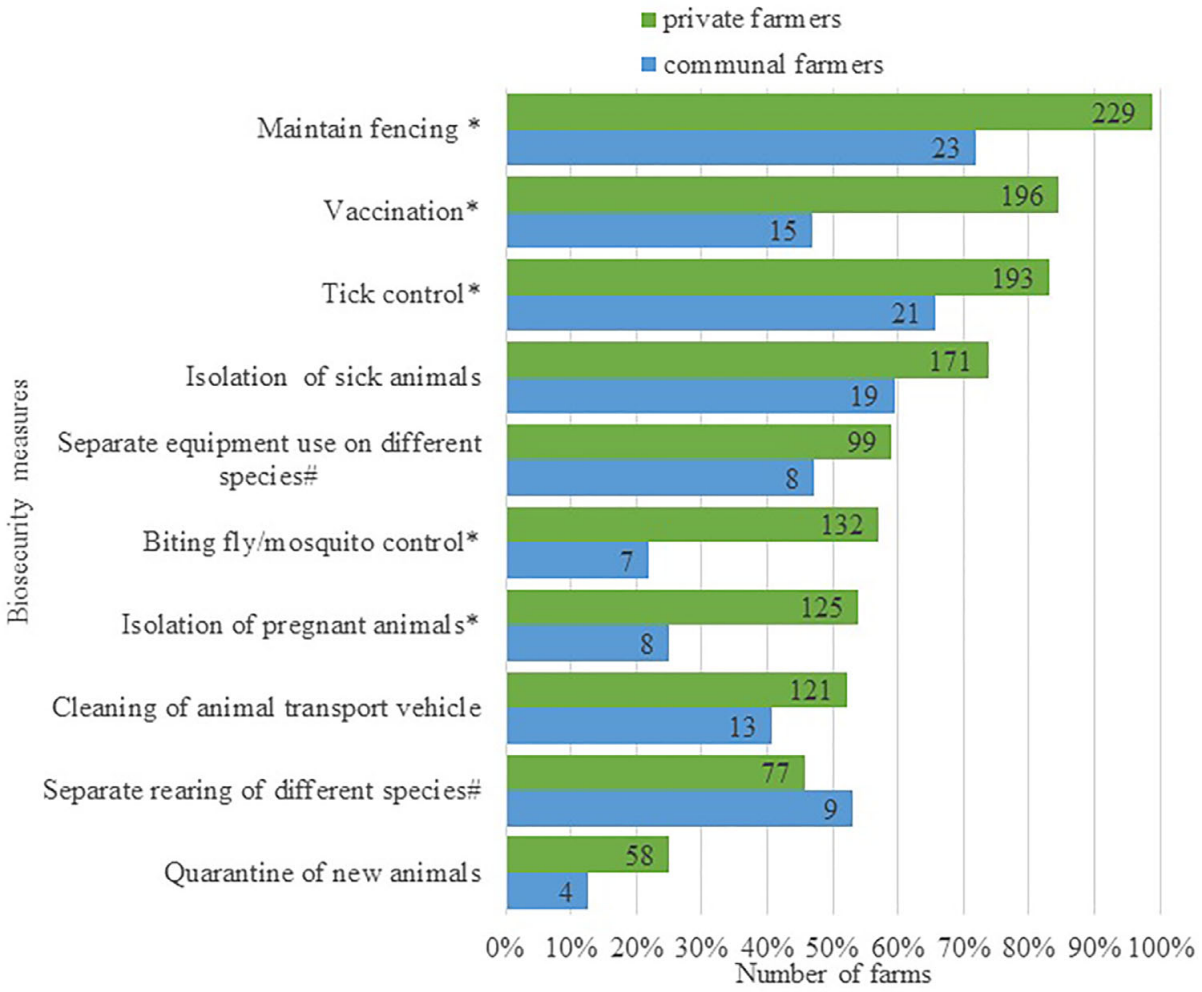
Biosecurity measures applied on ruminant livestock farms in the Free State and Northern Cape, South Africa. *Differed significantly between private and communal farmers. #Excluding farmers with one animal species only

Almost all farms, 99% (262 out of 264), used at least one of the 10 biosecurity measures evaluated (Figure 2). Private farmers implemented a median of 6 (IQR: 5–8; range: 0–10) biosecurity measures, whereas communal farmers used a median of 3.5 (IQR: 2–6; range: 1–9). The percentage and numbers of private and communal farms implementing each measure are shown in Figure 3.

Fences were present on nearly all private farms (99%; 229 out of 232), but not on communal farms (72%; 23 out of 32) (*p *< .001). Among private farms, 89% (203 out of 229) had wire mesh fencing around the property and 11% (26 out of 229) used electric fencing.

Among respondents, 84% (196 out of 232) of private farmers and 47% (15 out of 32) of communal farmers reported vaccinating their animals against any pathogen (*p *< .001). Thirty‐five percent (81 out of 232; 95% CI: 29–41%) of private farmers reported having vaccinated their animals against brucellosis. Only one communal farmer was aware that his livestock had received brucellosis vaccinations. Among private farmers that vaccinated against *Brucella* spp., the estimated median proportion of vaccinated animals was 100% (IQR: 70–100%; range: 3–100%; *n* = 77 of 81 farms with information). 38% (73 out of 193) of farmers vaccinated their cattle for *Brucella* spp., 8% (15 out of 180) vaccinated their sheep and 5% (3/63) vaccinated their goats. When asked when they had most recently vaccinated for brucellosis, 57 farmers reported vaccinating the year the survey was given (e.g., for the 2015 survey, the year was 2015), whereas 13 others reported earlier years with the earliest being in 2005.

Of private farms, 83% (193 out of 232) used control measures against ticks, whereas only 66% (21 out of 32) used them on communal farms (*p *= .03) (Figure 3). Fifty‐seven percent (132 out of 232) of the private farms sprayed to prevent mosquitoes or fly strike, whereas only 22% (7/32) of the communal farms did (*p *< .001). Farms that reported they applied acaricides did so a median of two times per year (IQR: 2‐4; range: 1–52). Overall, in the farms that reported seasonal tick control, acaricide treatments were primarily applied in the summer and/or autumn months (December–May on 22% of farms; 47 out of 212). Despite the fact that farmers were not asked about their knowledge and skills for using vaccine, tick and insect control measures, the survey team perceived this to be very variable in adequacy, but there is a need to look at the way measurements are applied in order to optimize them.

Private and communal farmers used various other biosecurity measures on their farms. Fifty‐nine percent (19 out of 32) of communal farmers reported isolating sick animals and 53% (nine out of 17) reared animal species separately. Less than half reported following other measures: 47% (eight out of 17) using separate equipment between species and 41% (13 out of 32) cleaning an animal transport vehicle (Figure 3). Fewer than 25% of communal farmers reported separating pregnant animals from the herd, or quarantining new animals prior to introducing them to the herd. In contrast, most private farmers (>50%) regularly used the following other measures: feeding, treating or working with sick animals after tending to the rest of the herd (*p *= .10), separating equipment by species (*p *= .83), isolating pregnant animals (*p *= .01) and cleaning animal transport vehicles (*p *= .26). Among private farmers, 46% (77 out of 168) kept different species separated (*p *= .69) (Figure 3). Only 25% of private farmers reported quarantining new animals (*p *= .18) (Figure 3).

### 3.6 Biosecurity measures used when introducing new stock

In the 12 months preceding the survey, 122 of the 264 farms had purchased animals. When introducing new stock into the herd, 34% (41 out of 122) of those farms used multiple biosecurity measures, 36% (44 out of 122) used a single measure and 30% (37 out of 122) did not use any. Vaccination of animals was the most commonly used measure, implemented by 57% (65 out of 115) of private farms and 43% (three out of seven) of communal farms, respectively, followed by use of acaricide for tick control, which was reported by only 25% (29 out of 115) of private farms. Only 22% (25 out of 115) and 14% (14 out of 115) of private farms reported quarantine and antibiotic administration, respectively, whereas single communal farms reported dipping, quarantine or antibiotic administration (Table 1).

**TABLE 1:** Biosecurity measures applied during the introduction of new animals on ruminant livestock farms in the Free State and Northern Cape, South Africa

Biosecurity measures for the introduction of new animals	Communal farmers (*n* = 7)	Private farmers (*n* = 115)
Vaccination	3 (43%)	65 (57%)
Dipping	1 (14%)	29 (25%)
Quarantine	1 (14%)	25 (22%)
Antibiotic administration	1 (14%)	14 (12%)
Other^a^	0	10 (9%)

^a^ Includes deworming, vitamin or traditional herbs supplementation as other practices they use for new animals.

### 3.7 Factors associated with implementation of biosecurity measures

Only three pairwise comparisons of biosecurity measures showed weak positive correlations between ‘separate rearing of different species’ and ‘separate equipment use of different species’ (φ = 0.4139), between ‘vaccination’ and ‘tick control’ (φ = 0.3611) and ‘tick control’ and ‘bite fly/mosquito control’ (φ = 0.3355). The negligible to low correlations between the biosecurity measures justified the assumption of independence for the binomial model (Table S1).

The variables ‘production system type’ and ‘land ownership’ were collinear as no communal farms classified themselves as having a feedlot or as being commercial, whereas 72% (168 out of 232) of the private farmers reported having a commercial business or feedlot. Therefore, only land ownership was used in the analysis. Similarly, variables regarding rearing of individual species were omitted from the analysis because they were used in the calculation of (and therefore collinear with) number of species reared.

**FIGURE 4:**
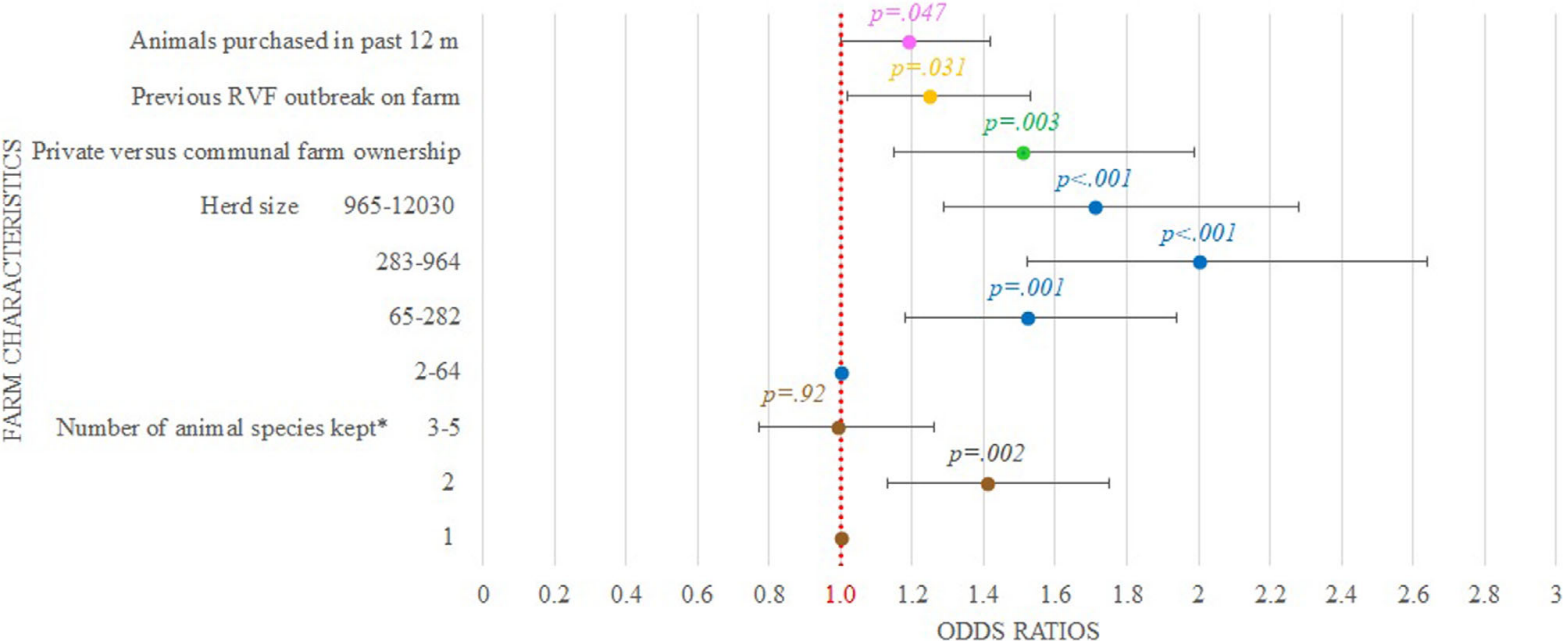
Factors associated and odds ratio (OR) values (coloured dots) and 95% confidence interval (black lines) for significant variables in the final maximum likelihood binomial model of using biosecurity measures on ruminant livestock farms (*n* = 264) in Free State and Northern Cape, South Africa. An OR of 1 is indicated by the red dotted line. *Cattle, sheep, goats, antelope, pig and horse

In the univariable analysis (Table 2), several farm characteristics were associated (*p *< .2) with biosecurity measure implementation and were selected for inclusion in the multivariable model (Figure 4).

**TABLE 2:** Univariable analysis of variables associated with implementation of biosecurity measures on ruminant livestock farms in Free State and Northern Cape, South Africa

		Number of biosecurity measures		
Variable	Number of farms	Median	IQR	*p* Value
Land ownership^a^				
Private	232	6	5–8	<.001
Communal	32	3.5	2–6	
Number of animals species reared^a^ (cattle, sheep, goat, pig, horse, antelope)				
1	86	4	3–6	
2	92	7	6–8	<.001
3	55	7	5–8	<.001
4	24	6.5	4–8	.01
5	7	7	6–8	.02
Animals mix with roaming wildlife^a^				
Yes	117	7	5–8	.01
No	147	6	4–7	
Animals were purchased in past 12 months^a^				
Yes	122	7	5–8	<.001
No	142	6	4–7	
Animals died in past 12 months				
Yes	146	6	4–8	.1
No	118	6	4–7	
Farm size (ha)^a^				
1–400	68	5	4–6.5	
401–1200	69	6	4–8	.04
1201–3000	63	6	5–8	.01
3001–15,000	58	6	4–8	.01
Herd size (Number of animals)^a^				
2–64	66	4	3–6	
65–282	66	6	4–7	<.001
283–964	66	7	6–8	<.001
965–12,030	66	7	5–8	<.001
Purpose of animal rearing^a^				
Meat	153	6	4–7	
Meat‐wool, wool	57	7	5–8	.02
Dairy/milk, dairy‐meat	12	7.5	4.5–8.5	.1
Resale, tourism, used in ceremonies, seen as wealth	42	4	3–6	<.001
Slaughtering was done on farm				
Yes	166	6	4–8	.7
No	98	6	4–7	
Animals aborted in past 3 months				
Yes	67	6	5–7	.96
No	197	6	4–7	
Brucellosis on farm in past^a^				
Yes	23	7	5–8	.1
No	241	6	4–7	
Rift Valley fever on farm in past^a^				
Yes	72	7	6–8	<.001
No	192	6	4–7	

^a^ Variable selected for inclusion in multivariable analysis *p *< .2

The following factors were associated with a greater odds of implementing of biosecurity measures in the final binomial regression model (Figure 4): farms that used private versus communal land (OR: 1.51; 95% CI: 1.15–1.99); farms that reared two animal species compared to only one species (OR: 1.41; 95% CI: 1.13–1.75) but not for three or more species (*p *= .92); farms with herd size of 65–282 animals (OR: 1.52; 95% CI: 1.18–1.94), 283–964 animals (OR: 2.00; 95% CI: 1.52–2.64) and 965–12,030 animals (OR: 1.71; 95% CI: 1.29–2.28), compared with <65 animals; farms that reported to have experienced RVF outbreaks previously (OR: 1.25; 95% CI: 1.02–1.53); and farms that had purchased animals in the 12 months prior to the survey (OR: 1.19; 95% CI: 1.00–1.42).

## 4 DISCUSSION

The majority of farmers implemented at least one biosecurity measure on their farm and many used more than six measures, which suggests that farmers in the study had awareness of some biosecurity measures. Other studies on European private farmers have found that, with the exception of some intensive commercial operations (primarily swine, poultry and ruminant feedlots), most rural livestock producers have a poor understanding of biosecurity (Bellini, 2018; Denis‐Robichaud et al., 2019).

A previous study conducted across five provinces in South Africa (including the Free State) among 532 smallholder farmers, defined as those who kept less than 100 livestock, found that 88% reported vaccination and 87% reported control of external parasites (Mdlulwa et al., 2021). Their findings were consistent with ours with respect to vaccination and tick control, despite the fact that our study included all farmers, only 33% of whom were smallholders. Furthermore, Mdlulwa et al.’s study (Mdlulwa et al., 2021) found that 26% of smallholder farmers isolated new or sick animals, and although our findings for quarantine of new animals (21%) agree with that figure, 72% of farmers isolated their sick animals from the rest of the herd.

Nearly all private farmers and most communal farmers reported using fencing around their farm because it provides physical security, such as confining farm animals, facilitating quarantine of new animals and keeping out unwanted animals and people that pose a threat of infectious diseases, predation or theft. We found that the vast majority of adult cattle and sheep were left overnight in the grazing areas on private farms likely due to the safety permitted by fencing, though the condition and maintenance of the fencing were not reported. In contrast, in communal areas, farmers reported keeping their livestock in a corral at night. Some farms did report predation losses. Reducing contact with wildlife is an important biosecurity measure as wild animals are involved in the epidemiology of many livestock and zoonotic diseases and may act as reservoirs for these pathogens (Kruse et al., 2004).

Vaccination was the second most frequently reported biosecurity measure. In contrast to our finding and despite the fact that multi‐pathogen vaccinations are the most cost‐effective way to prevent livestock disease, low vaccination rates have been reported amongst smallholder farmers in Africa, Asia and Latin America (Wallace et al., 2013; Donadeu et al., 2019). Rostal et al (2020) found that less than 60% of farmers vaccinated livestock during the RVF epidemic of 2010 in central South Africa, despite vaccination being the most effective measure to prevent loss of livestock and transmission to animal workers (Hartman, 2017).

Low to moderate vaccination levels for brucellosis were also reported by the farmers in our study, and cattle were vaccinated most frequently. Brucellosis is a reportable disease in South Africa as required by the Animal Diseases Act 35 of 1984 and the Bovine Brucellosis Scheme (R.2483 of 9 Dec 1988) (Department of Agriculture Forestry and Fisheries Republic of South Africa, 1984, 2017). All heifers 4–8 months of age are required to be vaccinated once. Currently, only high‐risk herds that have been confirmed or suspected of infection are required to test for bovine brucellosis. All other bovine herds are free to participate in the brucellosis testing programme (Department of Agriculture Forestry and Fisheries Republic of South Africa, 2017). Our results suggest that farmers may be aware of the threat of brucellosis, they likely find it difficult to clinically identify. As brucellosis control is a high national priority, the low vaccination rate reported in our study is cause for concern. However, it is possible that farmers who rely on government or private veterinary services may not always be aware of or remember the vaccinations their stock receive. The findings of low to modest vaccination use against diseases that were highly prevalent and had an impact in the area contrast with findings on increased willingness to invest in vaccination and other biosecurity measures when confronted with a threat (Merrill et al., 2019; Machalaba, 2020). This all suggests that there is still a lot of opportunity to discuss zoonoses control with farmers. More education is required about the risks of general and disease‐specific transmission, as well as the prevention of any practices that promote disease spread.

Farmers in our study reported using frequent and routine control measures for ticks. This is an important biosecurity measure with both economic and human health benefits (Jongejan et al., 2020) to prevent common tickborne diseases, such as heartwater in animals, and Q fever and Crimean‐Congo haemorrhagic fever in both animals and humans.

Isolation is a measure aimed at preventing the spread of disease from sick to healthy stock, but it must be used in conjunction with disinfection of facilities and working equipment to avoid fomite contamination in order to be effective (Bergström et al., 2012). Our findings show that most farmers are aware that sick animals can spread disease and that farmers have determined that isolating sick animals is an important precaution to protect the health of the remaining herd.

Farmers were much less likely to implement biosecurity measures when introducing new animals into the existing herd, despite the fact that the most common risk factor for the introduction of infectious diseases to a farm is the introduction of new animals (Cuttance & Cuttance, 2014).

Less than 60% of the farmers used vaccination when introducing new animals, compared with the much higher routine vaccination rate discussed earlier (up to 84%). This, combined with farmers reporting that they rarely cleaned and disinfected transport vehicles and did not routinely quarantine new animals (only 13% (16 out of 122) of the farms combined vaccination and quarantine), suggests that these farms are at risk for the introduction of infectious agents. Further, if a third party is used to move the animals and they also do not disinfect the transport vehicles, this may also pose a transmission hazard between farms. Livestock trade at both the local or regional level may contribute to disease spread (Fèvre et al., 2006).

Twelve percent of farmers reported administering antibiotics to animals upon introduction to the farm. Antibiotics are often used among introduced animals when other biosecurity/welfare measures on the farm are insufficient to prevent illness following the stress of transport. It is important for farmers to incorporate antibiotic stewardship when designing their biosecurity system to ensure their continued efficacy in both animal and human health (Landers et al., 2012; Chantziaras et al., 2014). Although antibiotics are as important for livestock health as they are for human health, this observed prudent use by livestock producers may be due to the farmers’ unwillingness to pay for additional biosecurity measures, or on the other hand may be a sign of good husbandry management; this requires more investigation. Vaccination and other non‐antibiotic interventions will help minimize the use of antibiotics within livestock populations, but this has not been without its difficulties (Hoelzer et al., 2018). We did not specifically inquire about animal deworming, but some of our farmers mentioned it as another practice they used. Antihelmintic and acaracide resistance, like antibiotic resistance, has become a significant problem in animal production as a result of inappropriate use; Therefore, farmers should be encouraged to invest in developing technical skills or receiving consistent veterinary services, as well as plan biosecurity strategies with veterinary practitioners in order to ensure that the measures implemented are adequate and effective (Hlatshwayo & Mbati, 2005). This also applies when administering any vaccine.

Private landowners likely implemented more measures because farmers who own the land would have more control over the territory as a resource, its condition and access by other farmers, making it easier to implement various biosecurity measures. For instance, only private landowners have a right to fence the farm. In contrast farmers using communal land would have less control over the territory as a resource, its condition and access by other farmers. Using communal land for farming restricts the farmer's ability to diversify their use of the land and expansion of their natural resource base, limiting livelihood improvement (Andrew et al., 2003). Given its history of colonialism and apartheid, South Africa's agrarian structure is still dominated by large‐scale farms (Neves, 2020), and because these farmers own the majority of the country's agrarian land, a large number of small‐scale farmers have small holdings or use communal land (Aliber et al., 2016). Furthermore, smallholders or communal farmers are often poorer than private large‐scale farmers, owing to difficulties in fully participating in formal livestock marketing, which may influence their decision to implement biosecurity measures (Sotsha et al., 2018).

Ruminant livestock farmers may find it more cost effective and beneficial to implement biosecurity measures in large herds, as described in poultry farms. A cost‐benefit analysis of poultry farms found that the average cost of biosecurity action is lower per animal in larger operations. This reduction was primarily due to lower labour costs per animal for biosecurity action (Siekkinen et al., 2008). Furthermore, commercial farmers with a larger herd of animals have higher sales returns and hence more financial resources to invest in farm operations, including farm biosecurity.

Farms with two species were likely to employ a broader range of biosecurity measures than single‐species farms, which may reduce their vulnerability to species‐specific diseases or susceptibility to diseases capable of inter‐species transmission. Furthermore, keeping two species may require different types of biosecurity, some of which are more effective with certain animals than others (Kalis et al., 2004; Scagliarini et al., 2012). Biosecurity in farms with more than two versus one species was however not significantly higher which could be because farmers keeping many species are usually less specialized and have poorer overall management, that is, there is confounding due to other unmeasured management variables.

Farmers are aware that introducing newly purchased animals to the herd poses a risk of infectious disease introduction to the farm, since farmers who had purchased animals in the 12 months prior to the study implemented more biosecurity measures than those who had not. However, as previously stated regarding vaccination, chemical use and quarantine by farmers, the combination of biosecurity measures, the varied level of technical skill applied by farmers and the timing and order in which they are used is important for effective prevention (Cuttance & Cuttance, 2014).

We discovered that when farmers were confronted with RVF outbreaks and were aware of the impact on their own farms, they were more likely to implement biosecurity measures, the most important of which is animal vaccination for RVF and that their motivation to engage in biosecurity measures improved, as reported by other studies (Merrill et al., 2019; Machalaba, 2020).

Mdlulwa et al. (2021) used partial proportional odds analysis to compare determinants for different biosecurity combinations implemented by South African smallholder farmers and found that they used a combination of supplementary feeding, vaccination and ‘biosecurity’ (FVB), mainly consisting of external parasite control or deworming, although sometimes including a few other measures (isolation of new/sick animals or restricted access). They indicated that household income, access to animal health facilities, contact with animal health technicians, farmer association and a positive attitude toward vaccination all had a positive impact the use of FVB. Although our study and that by Mdlulwa et al. (2021) differed in methodology, factors assessed, farmer population used, level of commercialization, land ownership and type of biosecurity measures evaluated, both findings suggest that biosecurity application is higher among those with better access to ‘resources’. Our descriptive analysis also found that commercial and feedlot farms implemented more biosecurity measures, though this could not be evaluated in the final model as it was collinear with land ownership. These findings were consistent with a study that found that intensive production systems implemented increased levels of biosecurity in Cameroon (Kouam et al., 2020). The variation in the use of biosecurity measures could also be due to a number of factors that were not assessed in our study, such as the costs, time and labour required to implement biosecurity; farmer priorities, education and socioeconomic differences; as well as a lack of evidence for the efficacy or suitability of specific measures for different production strategies (Oladele et al., 2013; Niemi et al., 2016; Denis‐Robichaud et al., 2019).

Further research exploring the reasons for the lack of, or variation in, implementation of certain biosecurity measures is imperative. In order to be persuaded of the need for biosecurity, farmers must have access to information on prevalent infectious diseases and their clinical course in animals, and the cost of having an outbreak, as well as on cost benefit of biosecurity measures especially in communal or pasture settings (Minjauw, 2000; Holleman, 2003; Mee et al., 2010; Damiaans et al., 2019). Rather than simply promoting biosecurity on its own, we recommend that this be supplemented by research to understand the prevalence of various diseases and discussions with farmers and veterinarians on how improved biosecurity can reduce exposures and improve productivity and market access. Resource prioritization is critical for farmers that face multiple economic threats. Individual farmers must choose which biosecurity measures they will implement based on the diseases with the greatest economic and/or health impacts, the feasibility of implementing the measures within their production system and the economic cost (Fast et al., 2015). It is important for the farmer to believe that the biosecurity measures are a good investment without perceiving the initial cost to be too high (Siekkinen et al., 2008).

## 5 CONCLUSION

Overall, this study provides important insights into current farm biosecurity practices, finding that most farmers do apply some biosecurity measures but also indicating widespread farm‐to‐farm variation in applied practices as well as some critical deficits. We found that land ownership, herd size and keeping two species of livestock, past outbreak experience, animal purchase all increased the likelihood of a farmer implementing more biosecurity measures. However, more research is required to gain a comprehensive understanding of what other characteristics are influential in determining or discouraging farmers from adopting biosecurity, and similar studies could be implemented in other areas. Increased biosecurity support would likely benefit all farmers, and the limited use of vaccination, tick control and isolation among communal or less commercialized farms implies that increased outreach and improved access to interventions are needed.

## CONFLICT OF INTERESTS

The authors of this manuscript declare no competing interests.

## ETHICS STATEMENT

The authors confirm that the ethical policies of the journal, as noted on the journal's author guidelines page, have been adhered to and the appropriate ethical review committee approval has been received. This study was part of the ‘Understanding Rift Valley fever (RVF) in the Republic of South Africa’ Project (URVFSA), implemented in the Free State and Northern Cape during 2014–2019 as a collaborative One Health study between EcoHealth Alliance, the National Institute for Communicable Diseases, University of Pretoria and several other partners. Ethical approval was obtained from Hummingbird IRB (no. 2014–25), US DTRA Research Oversight Board (CT 2014–33)/US Army Medical Research and Animal Materiel Command, Health Research Protections Office (A20745‐1.a,b), University of the Witwatersrand (M140306) and University of Pretoria (140/2018), and permission was given by the Free State and Northern Cape provincial Departments of Health (04/04/2015 and 2015/001) and the Department of Agriculture, Forestry and Fisheries (12/11/1/1/13) of South Africa.

## AUTHOR CONTRIBUTIONS

*Conceptualization*: M. K. R., V. M., C. C. and P. N. T.; *methodology*: V. M., C. C., P. N. T., S. T., M. K. R. and W. B.; *software*: M. K. R.; *validation*: P. N. T., M. K. R. and S. T.; *formal analysis*: V. M., P. N. T., S. T. and W. B.; *investigation*: V. M., C. C., C. M. and M. K. R.; *resources*: W. B. K.; *data curation*: M. K. R.; *writing—original draft preparation*: V. M.; *writing—review and editing*: all authors; *visualization*: V. M. and P. N. T.; *supervision*: P. N. T., M. K. R., S. T. and F. J. B.; *project administration*: M. K. R., C. C., W. B. K., J. T. P. and V. M.; *funding acquisition*: W. B. K., J. T. P. and M. K. R. All authors have read and agreed to the published version of the manuscript.

## Supporting information

SUPPORTING INFORMATION

SUPPORTING INFORMATION

SUPPORTING INFORMATION

## Data Availability

The data that support the study's findings and that can be disclosed per IRB protocol are available upon request from EcoHealth Alliance, 520 Eighth Ave Ste 1200, New York, NY 10018, from Melinda K. Rostal (rostal@ecohealthalliance.org).
