# Supplementary material for: Factors affecting the use of biosecurity measures for the protection of ruminant livestock and farm workers against infectious diseases in central South Africa
Source: Transbound Emerg Dis. 2022 Apr 5;69(5):e1899–912. doi: 10.1111/tbed.14525 (PMC9790579; doi:10.1111/tbed.14525)

2022/01/19 12:04:11 1

**Poisson Regression Power Analysis**

**Numeric Results when X1 is Binomial with Proportion = 0.5**

**And Phi (Over-Dispersion Parameter) = 1.0000**

**Mean R-Squared**

**Sample Response Baseline Exposure X1 vs Two-**

**Size Rate Rate Time Other X's Sided**

**Power (N) Ratio Exp(B0) (MuT) (R2) Alpha Beta**

0.80000 909 1.1000 5.0000 1.0000 0.2500 0.05000 0.20000

0.80000 246 1.2000 5.0000 1.0000 0.2500 0.05000 0.20000

0.80000 118 1.3000 5.0000 1.0000 0.2500 0.05000 0.20000

0.80000 71 1.4000 5.0000 1.0000 0.2500 0.05000 0.20000

0.80000 49 1.5000 5.0000 1.0000 0.2500 0.05000 0.20000

0.80000 36 1.6000 5.0000 1.0000 0.2500 0.05000 0.20000

0.80000 28 1.7000 5.0000 1.0000 0.2500 0.05000 0.20000

0.80000 23 1.8000 5.0000 1.0000 0.2500 0.05000 0.20000

0.80000 19 1.9000 5.0000 1.0000 0.2500 0.05000 0.20000

0.80000 17 2.0000 5.0000 1.0000 0.2500 0.05000 0.20000

**References**

Signorini, David. 1991. 'Sample size for Poisson regression', Biometrika, Volume 78, 2, pages 446-450.

**Report Definitions**

Power is the probability of rejecting a false null hypothesis. It should be close to one.

N is the size of the sample drawn from the population.

Exp(B1)/Exp(B0) is the response rate ratio due to a one-unit change in X1.

Exp(B0) is the response rate when all covariates have a value of zero.

Phi is the over-dispersion parameter used when the Poisson model does not fit.

R2 is the R-squared achieved when X1 is regressed on the other covariates.

Alpha is the probability of rejecting Exp(B1)/Exp(B0) is one.

Beta is the probability of accepting a false null hypothesis.

**Summary Statements**

A Poisson regression of a dependent variable of counts on a binary independent variable with

proportion = 0.5 using a sample of 909 observations achieves 80% power at a 0.05000

significance level to detect a response rate ratio of at least 1.1000 due to a one-unit change

in the IV. The baseline rate is 5.0000 and the mean exposure time is 1.0000. The sample size

was adjusted since a multiple regression of the covariate of interest on the other covariates

in the Poisson regression is expected to have an R-Squared of 0.2500.

2022/01/19 12:04:11 2

**Poisson Regression Power Analysis**

**Chart Section**


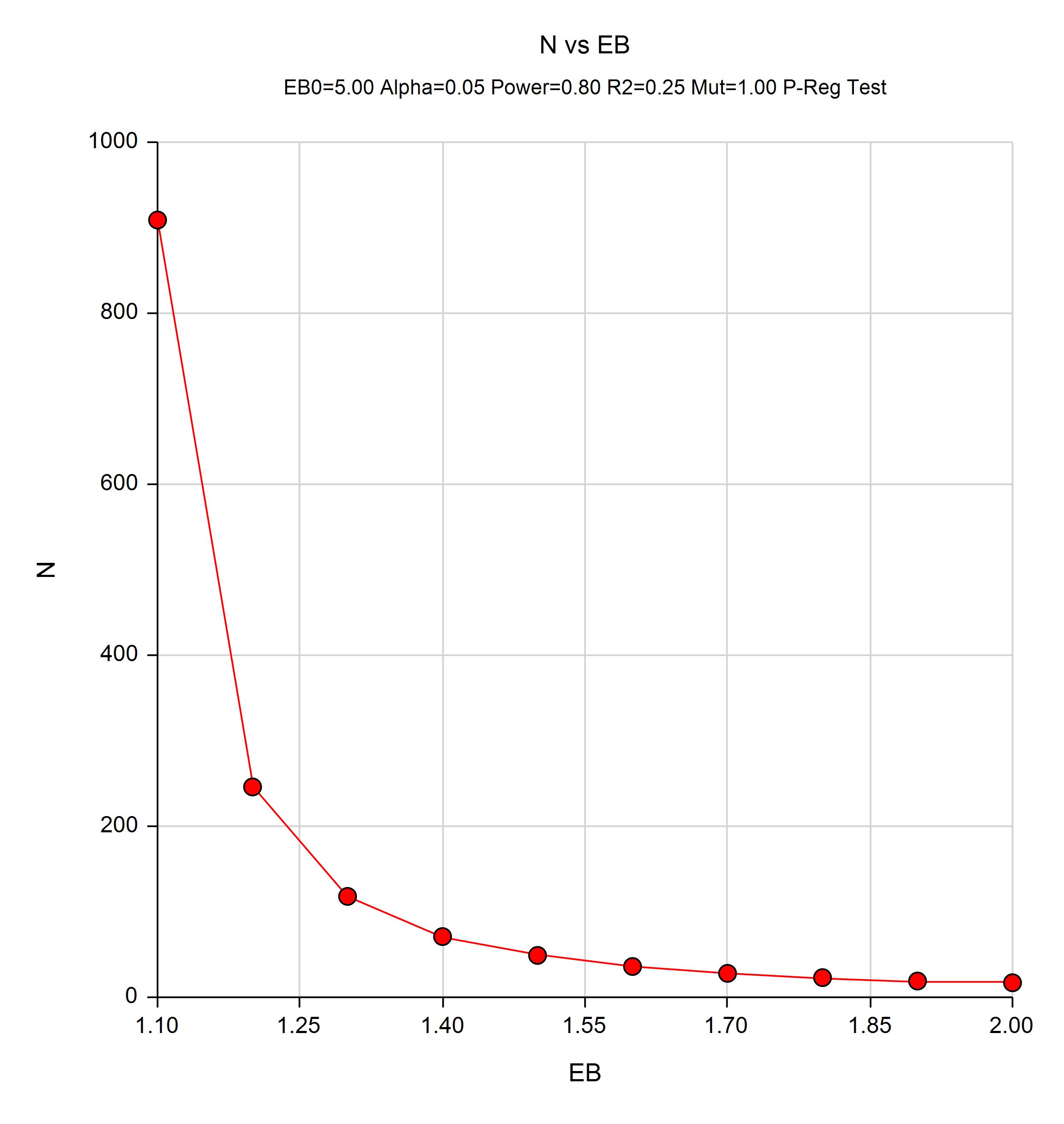

Supplement: Supplementary file 2 — SUPPORTING INFORMATION [file TBED-69-e1899-s003.docx]
